# Fluorescence Lifetime Correlation Spectroscopy (FLCS): Concepts, Applications and Outlook

**DOI:** 10.3390/ijms131012890

**Published:** 2012-10-09

**Authors:** Peter Kapusta, Radek Macháň, Aleš Benda, Martin Hof

**Affiliations:** 1 J. Heyrovský Institute of Physical Chemistry of ASCR, v.v.i, Dolejškova 3, 18223 Prague 8, Czech Republic; E-Mails: peter.kapusta@jh-inst.cas.cz (P.K.); radek.machan@jh-inst.cas.cz (R.M.); 2 Czech Technical University in Prague, Faculty of Biomedical Engineering, Sítná 3105, 272 01 Kladno, Czech Republic; 3 Centre for Vascular Research and Australian Centre for NanoMedicine, University of New South Wales, Sydney, NSW 2052, Australia; E-Mail: a.benda@unsw.edu.au

**Keywords:** fluorescence correlation spectroscopy (FCS), time correlated single photon counting (TCSPC), fluorescence cross-correlation spectroscopy (FCCS)

## Abstract

Fluorescence Lifetime Correlation Spectroscopy (FLCS) is a variant of fluorescence correlation spectroscopy (FCS), which uses differences in fluorescence intensity decays to separate contributions of different fluorophore populations to FCS signal. Besides which, FLCS is a powerful tool to improve quality of FCS data by removing noise and distortion caused by scattered excitation light, detector thermal noise and detector after pulsing. We are providing an overview of, to our knowledge, all published applications of FLCS. Although these are not numerous so far, they illustrate possibilities for the technique and the research topics in which FLCS has the potential to become widespread. Furthermore, we are addressing some questions which may be asked by a beginner user of FLCS. The last part of the text reviews other techniques closely related to FLCS. The generalization of the idea of FLCS paves the way for further promising application of the principle of statistical filtering of signals. Specifically, the idea of fluorescence spectral correlation spectroscopy is here outlined.

## 1. How Does FLCS Work?

FLCS is a variant of fluorescence correlation spectroscopy (FCS) [1,2] which uses differences in fluorescence intensity decays to obtain separate FCS autocorrelation functions (ACFs) of individual fluorophore populations in a mixture. The separation is performed by weighting each photon with a statistical filter function during calculation of the ACFs. Since the idea of statistical filtering is clearly explained elsewhere [3] and also outlined in other review articles [4,5], we will provide only a brief explanation here.

To use the fluorescence lifetime information in FCS, the arrival time of the photon has to be known on two different time-scales: (i) lifetime-scale (with picoseconds resolution), which measures the time between photon arrival and the respective excitation pulse and (ii) FCS-scale (microsecond resolution at least), which measures the time between photon arrival and the beginning of the experiment. Thus, the primary FLCS data output is a list of photon records containing two timing figures.

The photons are usually detected by time correlated single photon counting (TCSPC), where the arrival time on the lifetime-scale is expressed in units of TCSPC channel number *j*. For example when a 50 ns interval between consecutive excitation pulses is digitized into 1000 channels, the resolution of the lifetime-scale is 50 ps. The timing *t* on the FCS-scale is typically measured in units of excitation cycles; for example in the case of 20 MHz excitation repetition rate, the resolution is 1/20 MHz = 50 ns.

By sorting the recorded photons according to their channel number *j*, we obtain a histogram *I_j_*, which represents the total fluorescence decay curve. Let us now assume a sample containing *M* decay components indexed as *k* = 1…*M*. In that case *I_j_* can be expressed as a linear combination of individual decay patterns *p_j_*^(^*^k^*^)^, each multiplied by the number of photons *w*^(^*^k^*^)^ contributed by the respective component.

(1) $$I_{j}=\sum_{k=1}^{M}w^{\left(k\right)}{p_{j}}^{\left(k\right)}$$

The decay patterns *p_j_* can be obtained either experimentally (as fluorescence intensity decays of individual components measured separately) or by mathematical decomposition of the total intensity decay *I_j_* into a sum of mono- or multi-exponential decay functions assumed for the individual components. The following definitions of ACFs illustrate the difference between a standard FCS ACF (Equation 2a) and a filtered ACF in FLCS (Equation 2b).

(2a) $$G(\tau )=\frac{\langle I(t)I(t+\tau )\rangle }{{\langle I(t)\rangle }^{2}}-1$$

(2b) $$G^{\left(k\right)}(\tau )=\frac{\langle \sum_{j}{f_{j}}^{\left(k\right)}I_{j}(t)\sum_{j}{f_{j}}^{\left(k\right)}I_{j}(t+\tau )\rangle }{{\langle \sum_{j}{f_{j}}^{\left(k\right)}I_{j}(t)\rangle }^{2}}-1$$

Pointed brackets indicate averaging over all values of time *t* (measured on the FCS-scale). The so called filter functions *f_j_*^(^*^k^*^)^ are calculated by straightforward matrix algebra from the total intensity decay *I_j_* and decay patterns *p_j_*^(^*^k^*^)^ as derived elsewhere [6,7].

While in standard FCS each photon contributes equally to the ACF (its weight is one), the situation in FLCS is different. A single photon contributes to FLCS ACF of the *k*-th component with a certain weight depending on the photon’s TCSPC channel number *j*. The weight is given by the corresponding filter function *f_j_*^(^*^k^*^)^; its absolute value can be larger than 1 and its sign can be even negative. Nevertheless, the sum of filter function values for all *M* components equals 1 at any TCSPC channel *j*, that means $\sum_{k=1}^{M}{f_{j}}^{\left(k\right)}=1$. This is necessary in order to conserve the total number of photons entering calculation of ACFs. The characteristic features of FLCS filter functions are explained in an intuitive manner by Kapusta *et al*. [3].

## 2. A Brief History of FLCS

FLCS was invented in 2001 by Jörg Enderlein, who was working at that time in Forschungszentrum Jülich, Germany. That year, he and his co-workers have submitted a manuscript to Chemical Physics Letters about a “new method of performing FCS measurements for mixtures of several fluorescent molecular species” which “uses time-resolved fluorescence detection for separating the different FCS contributions from the different species”. The paper titled “Time-resolved fluorescence correlation spectroscopy” appeared in February 2002 [6] and contains not only the mathematical foundations of the method, but furthermore, a set of proof-of-concept experiments including details of the hardware implementation. Despite the very promising features, the proposed method did not receive appropriate attention, perhaps due to ongoing boom of biophysical applications of conventional FCS back then.

FLCS (still called time-resolved FCS or TCSPC-FCS that time) enters the scene again in 2005. In reference [7], Enderlein and Gregor describe the simplest and one of the most useful applications of FLCS: quick and easy removal of the distorting effect of detector after pulsing. The same year Benda *et al.* [8] showed how to upgrade a commercial FCS microscope (ConfoCor 1 from Zeiss) to be FLCS capable and demonstrate the first cross-correlation measurement using the perfectly overlapping “virtual” confocal volumes in a single colour FCS experiment.

To our knowledge, the first paper using the term FLCS is reference [9]. Since year 2006 the method can be considered established and the biophysical applications published up to now are reviewed in the next chapter. An introduction to the basic principles for beginners, together with recipes how to actually perform FLCS experiments and calculations has been published in reference [3]. The same paper contains also hardware details and demonstrates the usefulness of the simplest FLCS procedures. Thorough benchmark tests finally appeared in references [10,11] that demonstrate remarkable features unattainable by conventional FCS, for example resolution of diffusion time differences as low as 25% and separation of four signal components.

We are focusing here on an overview of, to our knowledge, all FLCS studies published since the first appearance of the method in 2001. Although the number of studies based on applications of FLCS has not yet exceeded 20, they form a relatively representative collection demonstrating a variety of possible applications of FLCS and can inspire other FCS users to include FLCS in their work. We believe that FLCS will become more widespread in the near future and has the potential to become a standard method for many applications especially thanks to growing availability of suitable instrumentation. Last but not least, we mention other techniques related to FLCS and generalization of the idea of statistical filtering, which is the core principle behind FLCS. Recent works suggest that the idea is more general and, besides fluorescence decay patterns, it can work with fluorescence spectra for example and in the future may also find other applications besides processing of FCS data.

## 3. Applications of FLCS

FLCS has been always compared to dual- or multi-colour FCS [6] for its ability to obtain separately autocorrelation functions of individual components of a mixture. It is, therefore, not surprising that FLCS has found applications similar to those of dual-colour FCS and fluorescence cross-correlation spectroscopy (FCCS) [12,13]. Padilla-Parra *et al.* have used FLCS to avoid spectral crosstalk in dual-colour FCCS in living cells [12]. They applied pulsed excitation at 470 nm (GFP) simultaneously with a continuous wave (CW) excitation at 561 nm (mCherry) and combined spectral separation of signals (by means of a dichroic mirror and optical filters) with FLCS filtering based on different TCSPC patterns (exponentially decaying in the case of GFP and uniform in the case of mCherry); the FLCS filtering eliminates spectral bleed through of GFP fluorescence into the detection channel of mCherry fluorescence and vice versa and thus, it eliminates false cross-correlation resulting from the spectral bleed through without a need for tedious corrections [14]. A similar goal can be achieved by using pulsed interleaved excitation [15], which requires however a pair of pulsed excitation lasers. Since economically priced pulsed laser diodes are not available in the spectral range optimal for excitation of mCherry or mRFP, the FLCS based approach is an interesting alternative.

Chen and Irudayaraj [13] based their cross-correlation study in living cells fully on FLCS principles using excitation at a single wavelength and pairs of fluorophores with overlapping spectra but different lifetimes (GFP and Alexa-488). By using FLCS with single wavelength excitation instead of dual-colour excitation, they not only fully avoided spectral crosstalk but furthermore, the artefacts resulting from nonideal overlap between the effective detection volumes corresponding to the individual excitation wavelength [13]. Nevertheless, care is needed in obtaining FLCS cross-correlation functions (CCFs), due to possible artefacts as discussed in section 4.4.

The analogy between FLCS and dual- or multi-colour FCS has, however, only limited validity. Besides applications analogous to dual-colour FCS and FCCS, FLCS offers other unique possibilities. One of its main advantages lies in the possibility for obtaining separately ACFs corresponding to fluorophores in different environments or different molecular states (e.g., conformational or protonation states). While environmental spectral shifts are typically too small to be used in dual-colour FCS, the corresponding changes in lifetime are sufficient for the use in FLCS [9]. The first works on applications of FLCS have already focused on the possibilities of separating ACFs from identical fluorophores in two different environments or states and so has, actually, the majority of all published applications of FLCS.

In 2006 Benda *et al*. [9] described a method to separate FCS signals from supported lipid bilayers (SLBs) and from the aqueous phase by using FLCS and so-called lifetime tuning. The authors addressed a problem commonly encountered in all FCS studies of diffusion in lipid membranes of molecules with non-negligible partitioning to the aqueous phase. Since the lipid bilayer has a thickness in the order of nanometers, it occupies only a small part of the effective detection volume and, therefore, fluorescent molecules present in the aqueous phase are considerably contributing to the FCS signal. This complicates the determination of lateral diffusion coefficients of membrane-associated molecules as well as the determination of their partitioning in the lipidic phase. Benda *et al.* exploited quenching of fluorescence in the vicinity of light-absorbing materials (such as silicon and indium-tin oxide) [9]; fluorophores associated with SLBs adsorbed on such light-absorbing surfaces are quenched and their fluorescence lifetime is considerably shorter than of fluorophores in the aqueous phase. Therefore, separate autocorrelation functions of fluorophores in the aqueous phase and of the SLB-associated fluorophores can be obtained by FLCS. Since the quenching does not only shorten the lifetime but also reduces brightness of the SLB associated fluorophores, a compromise has to be found between the requirements of large difference in fluorescence lifetime and of high molecular brightness (needed for all FCS techniques); the efficiency of quenching can be tuned by introducing layers of dielectric spacers (such as silicon dioxide or oriented lipid multilayers) between the SLB and the quenching surface [9].

A similar application of FLCS was reported in 2008 [16] in which shortening of lifetime of a fluorophore on the surface of silver nanoparticles was utilized. Single stranded oligonucleotides were covalently attached to the nanoparticles and then hybridized with fluorescently labelled complementary oligonucleotides. The main advantage of the silver nanoparticles was their ability to enhance fluorescence intensity, thanks to which they can be investigated by FCS even in the presence of excess of free fluorophore [16,17].

The two above mentioned studies are of purely methodological character and although they suggest perspective applications of FLCS, they do not address any issue of actual importance for biological or molecular sciences. The work on DNA compaction published by Humpolíčková *et al.* in 2008 can be in that sense considered the first applications of FLCS [18–20]. Compaction of DNA by cationic compounds occurs naturally in nuclei of cells and is also highly relevant for non-viral gene therapy to aid the transport of large charged DNA molecules across the plasma membrane. While the detailed molecular mechanism of compaction of large DNA molecules can be directly observed by fluorescence microscopy [21,22], FLCS contributed significantly to understanding of molecular details of DNA compaction by elucidating the compaction of DNA molecules smaller than diffraction limited resolution (10 kbp [18–20,23]). DNA molecules were labelled by PicoGreen^®^ (Invitrogen, Carlsbad, CA, USA), a fluorescent dye which intercalates between DNA bases and its lifetime changes from approximately 4.2 ns (in uncondensed DNA) to approximately 3.5 ns (in fully condensed DNA) [20]. Comparison of ACFs of uncondensed and fully condensed DNA, respectively, measured separately with ACFs of individual lifetime components extracted from the FLCS measurement in the midpoint of titration (of the DNA sample with the condensing agent) revealed that compaction by spermine (a multivalent cation) is a discrete process (DNA molecule is either in uncondensed or in fully condensed state) [18,20]. On the other hand, compaction by cetyltrimethylammonium bromide (a cationic surfactant) was shown to proceed gradually [18]. Analysis of cross-correlation between the individual lifetime components in the midpoint of titration by spermine indicated dynamic transitions between condensed and uncondensed state on millisecond timescale [19]. The first of the studies [18] is also innovative from the point of view of methodology of FLCS, demonstrating how FLCS can be used in cases when neither decay patterns nor fluorescence lifetimes of both components in the mixture can be measured separately. Such situations are common, when only the decay pattern of one of the components can be measured separately, while the other component exists only in mixture of both components (due to establishment of chemical equilibria). It is possible to use FLCS in such cases to filter out the contribution of the component with known decay pattern and obtain ACFs corresponding to the remaining component only.

The previously mentioned study [19] illustrates a frequently used feature of FLCS: the possibility to characterize rate constants of transitions of the fluorophore between the states corresponding to individual lifetimes. It is worth noting that such information can be obtained thanks only to the ability of FLCS to distinguish signal from the same fluorophore in different states; no analogous experiment based on multi-colour FCS does exist. When transitions between the states occur, they are manifested in ACFs of each individual component in the same manner as transitions to dark states [24]. Assuming that the fluorophore can exist in the sample in two states, A and B are characterized by different lifetimes; assuming no other dark states, the decays of the ACFs are, then, determined by translational diffusion of the molecules and by transitions between states A and B. The ACF for the component A is described by Equation 3, where *F_B_* is the average fraction of molecules in state B (the dark state in this case) and *τ_P_* is the reciprocal value of the sum of rate constants of transitions from state A to B and from B to A [24].

(3) $$G(\tau )=\left[1-F_{B}+F_{B}\;{\text{e}}^{-\frac{\tau }{\tau_{P}}}\right]\frac{1}{N(1-F_{B})}\frac{1}{1+(\tau /\tau_{D})}\sqrt{\frac{1}{1+(\tau /\tau_{D}){\left(\omega_{0}/\omega_{z}\right)}^{2}}}$$

This approach has been used by Paredes *et al.* to study proton-transfer reactions of a fluorescein derivative Tokyo Green-II (TG-II) in different environments [24–26]. TG-II exists in two forms: protonated (shorter lifetime), which is neutral at physiological pH and deprotonated (longer lifetime), anionic at physiological pH. The equilibrium and the kinetic constants of the transitions depend on the pH. The authors show that the rate constants of the transitions are influenced by species acting as proton donors or acceptors. The influence of several buffers and salts was studied and it was shown that the rate constants in the presence of different cations [24] and anions [26] are ordered according to Hofmeister series, the rate constants being higher in the presence of kosmotropic than in the presence of chaotropic ions. Similarly it has been shown that Ficoll, a polymer used as a noninteracting crowding agent to mimic intracellular environment, forms a surface complex with TG-II and influences rate constants of the transition between neutral and anionic state of the dye [25]. Orte *et al.* used FLCS and TG-II dye to study reversed micelles of surfactant sodium bis(2-ethylhexyl) sulfosuccinate in organic solvents [27]. The reversed micelles are able to encage nanopools of water in their interior, in which TG-II was dissolved. The FLCS experiment provided at the same time diffusion coefficients of whole reversed micelles, indicating the size of the micelles formed, as well as information on kinetics of transitions between anionic and neutral form of the dye. The transitions between the two forms of the dye are much faster than in bulk water due to high concentration of proton donors and acceptors at the interface between water nanopool and surfactant polar heads [27].

More comprehensive information on dynamic transitions between the states corresponding to individual lifetimes can be extracted from CCFs in FLCS; transitions between the states are manifested by rising terms in the CCFs, which contain information on the timescale of the transitions. This possibility has been already used in the study of mechanism of DNA compaction [19]. A detailed discussion of the analysis of CCFs in FLCS is provided in the work of Gregor and Enderlein [28] who demonstrate the method on experiments with streptavidin labelled by a cyanine dye (Cy5). The complex can exist in two different fluorescent states which differ in fluorescence lifetime; the rate constants of transitions between the two states can be extracted from FLCS CCFs.

Analysis of ACFs of individual components and of CCFs between them was recently used to extract kinetic rates of transition of a protein (Syntaxin 1) between its two conformational states [29]. Each protein molecule was labelled by Alexa488 (donor) and Alexa594 (acceptor); in the closed conformation the donor is located closer to the acceptor than in the open conformation and its lifetime is, thus, shorter. Two mono-exponential decays with lifetimes 3.6 ns and 0.8 ns for the open and closed conformation respectively were used to calculate the FLCS filters. It is, to our knowledge, the first application of FLCS using Förster resonance energy transfer (FRET) as the source of lifetime difference; such approach has numerous potential applications in biomolecular research. Apart from that the study is interesting from methodological point of view for using polarization resolved detection as a part of multiparameter detection concept denoted filtered FCS (fFCS) by the authors (see section 5 for more details).

**Figure 1:**
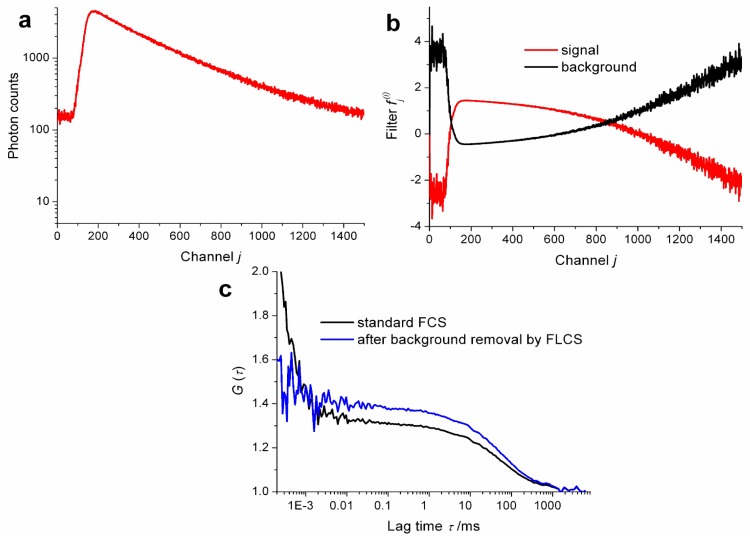
Illustration of removal of background-related artefacts in autocorrelation functions *G* (τ) by Fluorescence Lifetime Correlation Spectroscopy (FLCS). (**a**) The time correlated single photon counting (TCSPC) histogram of photon detection times (each channel corresponds to 16 ps); uniformly distributed background caused by detector after pulsing, thermal noise or photons of stray light adds over 100 counts to each channel. (**b**) FLCS filters calculated for fluorescence signal (red) and for the uniform background (black). (**c**) Comparison of autocorrelation function calculated without (black) and with FLCS filtering (blue); the decay on μs timescale is caused by detector after pulsing, while the lowering of autocorrelation amplitude is a result of uncorrelated background (thermal noise, stray light). The data were measured in a supported lipid bilayer on glass containing fluorescently labelled lipid as a tracer for lipid diffusion. More details on the experiment can be found in reference [32].

Apart from its ability to extract ACFs of individual species in a mixture and CCFs between them, FLCS is an excellent method to purify FCS data from parasitic signal components such as dark counts and after pulsing of the detector or photons of elastic and Raman scattering [3,7,10,11,30]. The removal of dark counts and after pulsing by FLCS is especially simple and straightforward (a single detector is sufficient and no a priori knowledge of the decay pattern is needed) and at the same time has large impact on quality of FCS data. See Figure 1 for an illustration of the influence of background on ACFs. The approach has been, therefore, accepted by several researchers as a standard part of FCS data analysis and is being used without explicit reference to the FLCS method [31–33]. We have, therefore, no estimate of how widespread the use of FLCS for background suppression is; nevertheless, we believe it has the potential to be by far the most widespread application of FLCS.

## 4. Frequently Asked Questions about FLCS

### 4.1. What Hardware Do I Need?

Basic requirement is a confocal microscope setup capable of conventional FCS measurement. There are several commercial products directly supporting FCS; most contemporary confocal laser scanning microscopes can also be upgraded for F(L)CS capability [34]. At least one picosecond pulsed laser source is necessary. Thanks to the variable pulse repetition rate, gain switched laser diodes are very well suited. The requirements for pulse duration (below 1 ns, ideally some tens of ps) and pulse energy (typically picojoules) are relaxed. However, the repetition rate should be on the order of megahertz due to TCSPC involved in detection. Multiphoton excitation based e.g., on mode-locked titanium-sapphire laser can be utilized, too. In FCS, high photon detection efficiency is a fundamental requirement. In practice, single photon avalanche diodes (SPADs) or special photomultiplier tubes (PMTs) operated in photon counting mode are employed. The output of these detectors (an electronic pulse for each detected photon) should be directly accessible and connected to a timing device capable of measuring and storing pulse arrival times on two different time-scales as specified in section 1.

From FLCS point of view the key performance parameters of the timing electronics are short dead time (high throughput), low differential non-linearity (ripple- and distortion-free histograms), time base stability (truly linear time axis) and time-resolution. As of today, there are two suppliers of such timing devices with sufficient quality: PicoQuant GmbH (product names: TimeHarp 200, PicoHarp 300 and HydraHarp 400, Berlin, Germany) and Becker & Hickl GmbH (SPC-series boards, Berlin, Germany). The vast majority of published FLCS work was done with PicoQuant devices, because the company offers complete FLCS capable confocal systems, including the necessary software (SymPhoTime, version 5.3.2, PicoQuant, Berlin, Germany, 2010).

### 4.2. What Is the Smallest Lifetime Difference that Allows Separation of Components in FLCS?

The quick and surprising answer is “zero”. For a rigorous answer one has to define what is meant exactly by lifetime.

It is obvious that the larger the (decay) lifetime difference, the easier is to resolve two components from each other. Signal components featuring strictly single exponential decays with, say, nanosecond lifetimes differing only by 0.1 ns are practically irresolvable from each other. This is true even for the best TCSPC setups and a simple cuvette format experiment when the sample is prepared so that the fractional intensities of these two emissions are equal. In order to resolve two such similar lifetime patterns from each other, a tremendous amount of photons (that is: extreme high quality decay curve) has to be collected.

**Figure 2:**
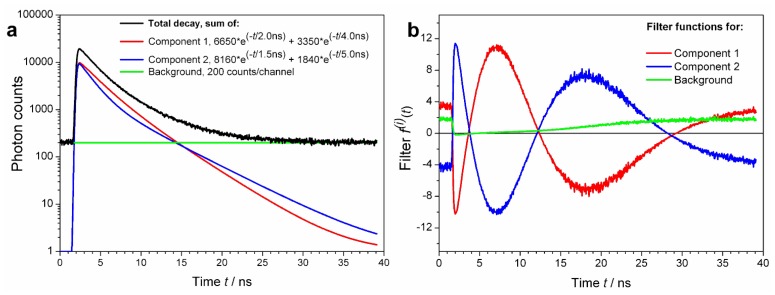
Simulated, realistic decay curve, its components and their calculated FLCS filter functions. (**a**) Two bi-exponential decay functions with the indicated amplitudes and lifetimes were convoluted with a real instrument response function (IRF not shown) resulting in Components 1 and 2, respectively. Both components have an average lifetime of 3.00 ns, in spite of different shapes. Uniform background (200 counts in each TCSPC channel) is added. The total decay curve is an arithmetic sum of these three components, plus the Poisson noise. (**b**) Calculated filter functions for decay components of the Total decay curve. Although the goal is to resolve Component 1 from Component 2, finding and subtracting the decay background (the third “decay” pattern) is so trivial that it always should be included in the filter calculation. The advantage is that the resulting ACFs for Components 1 and 2 will be purged of detector after pulsing and uncorrelated background contributions.

However, most fluorophores in real life samples do feature multi-exponential decays. It is common to characterize them by a single average lifetime. In FLCS, it is not the lifetime, but the decay shape (pattern) what matters. Thus, it is possible to resolve two signal components with exactly the same average lifetime, provided their TCSPC patterns are sufficiently different. An example of such a case is shown using simulated data in Figure 2. Consider two fluorescence decays:

$$\begin{array}{l} \text{Component }1\;(t)=6650\cdot \text{exp}(-t/2.0\;\text{ns})+3350\cdot \text{exp}(-t/4.0\;\text{ns})\;\text{and}\\ \text{Component }2\;(t)=8160\cdot \text{exp}(-t/1.5\;\text{ns})+1840\cdot \text{exp}(-t/5.0\;\text{ns}) \end{array}$$

These are plotted in Figure 2a as Components 1 and 2, respectively. Both decays have the same intensity weighted average lifetime *τ_AV_* of 3.00 ns, calculated as *τ_AV_* = (∑*A_i_*·*τ_i_*^2^)/(∑*A_i_*·*τ_i_*), *i* = 1, 2. Yet, the two shapes are clearly different, visible by bare eyes. The filter functions (weights) do not reach extreme values that would lead to noisy ACFs. Calculation of decay specific ACFs using the filters shown in Figure 2b would be easy provided enough (more than 10^6^) photons are collected in the FLCS experiment.

**Figure 3:**
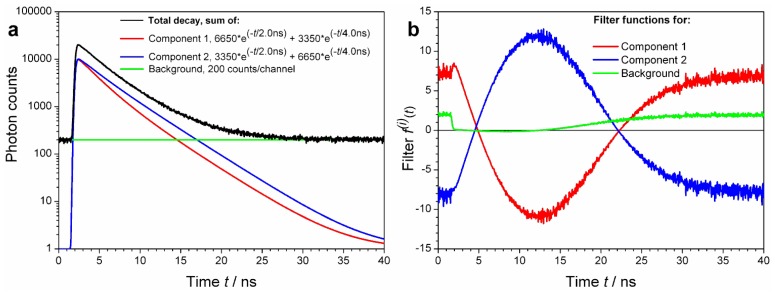
Simulated, realistic decay curve, its components and their calculated FLCS filter functions. (**a**) Two bi-exponential decay functions with the indicated amplitudes and lifetimes were convoluted with a real instrument response function (IRF not shown) resulting in Components 1 and 2, respectively. They have different average lifetimes, 3.00 ns and 3.60 ns, respectively, in spite of the same lifetime constituents. Uniform background (200 counts in each TCSPC channel) is added. The total decay curve is an arithmetic sum of these three components, plus the Poisson noise. (**b**) Calculated filter functions for decay components of the Total decay curve. Although the goal is to resolve Component 1 from Component 2, finding and subtracting the decay background (the third “decay” pattern) is so trivial that it always should be included in the filter calculation. The advantage is that the resulting autocorrelation functions (ACFs) for Components 1 and 2 will be purged of detector after pulsing and uncorrelated background contributions.

Yet another example is shown in Figure 3a, where the two decay (signal) components have exactly the same lifetime content:

$$\begin{array}{l} \text{Component }1\;(t)=6650\cdot \text{exp}(-t/2.0\;\text{ns})+3350\cdot \text{exp}(-t/4.0\;\text{ns})\;\text{and}\\ \text{Component }2\;(t)=3350\cdot \text{exp}(-t/2.0\;\text{ns})+6650\cdot \text{exp}(-t/4.0\;\text{ns}) \end{array}$$

Despite of the identical lifetime components, owing to different associated amplitude values the decay patterns (characterized by intensity weighted average lifetimes) of these two decays are different: *τ_AV_*_1_ = 3.00 ns and *τ_AV_*_2_ = 3.60 ns, respectively. Again, calculation of decay specific ACFs is possible.

In this context, it is obvious why fluorescence anisotropy dynamics is a well suited molecular property to be utilized in FLCS [29]. Dynamic anisotropy manifests itself as an additional fast (multi-) exponential rising or decaying component of the intensity decay curve. Therefore, it is influencing the onset of the decay where the intensity is the highest. In other words: many photons are affected by anisotropy, which has a significant effect on the FLCS filter curves, facilitating the filtering.

### 4.3. What Is the Smallest Difference of Diffusion Constants that Can Be Resolved by Means of FLCS?

The answer is again “zero”. Resolving two diffusing species (in effect: two ACFs) by means of FLCS is based on TCSPC, that is: based on nanosecond/picosecond fluorescence decay behaviour, NOT on measuring focus transit times (diffusion times). Thus, if two species in the sample have sufficiently different decay signatures (patterns), their ACFs can be separated, regardless of their equal diffusion constants. The calculated filtered ACFs of such components will reveal identical diffusion times, however, the time-zero amplitude of those ACFs (corrected for possible triplet or other fast ACF decay contributions) will be proportional to the inverse of the component concentrations.

### 4.4. Are CCFs in FLCS Free of Artefacts Encountered in Dual-Colour FCCS?

The simple answer is “yes”. FLCS is free of the two main sources of artefacts in dual-colour FCCS: the spectral bleed through and non-ideal overlap of excitation and detection volumes for the two fluorophores [14]. The latter is true only if both fluorophore populations in the FLCS experiment have completely overlapping emission spectra and differ in fluorescence decay solely; FCCS using single excitation wavelength (and thus identical excitation volume for both fluorophores) has shown that the differences in detection volumes are sufficient to introduce significant artefacts into CCFs [35].

However, unfortunately, that does not mean that CCFs in FLCS are absolutely free of artefacts. Physically unsubstantiated negative CCFs amplitudes (anticorrelation) were observed by some authors [36] and also reported in literature [29]. There are several factors which may contribute to the observed anticorrelation; we discuss here three of them.

**Figure 4:**
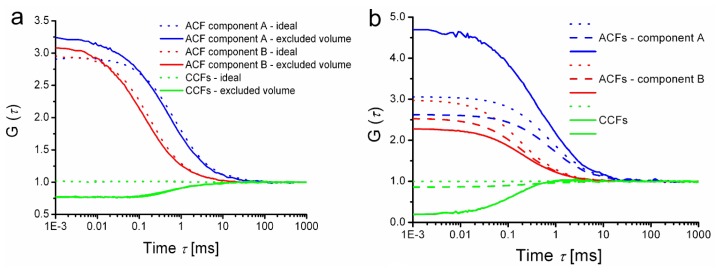

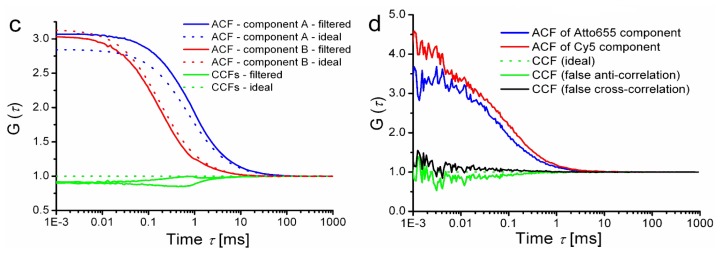
Illustration of possible sources of anticorrelation in FLCS. (**a**) to (**c**): Two-dimensional diffusion was simulated in a 6 μm × 6 μm square simulation box with periodic boundaries with 1 μs step; the Gaussian detection area had a radius of 240 nm; the simulated sample contains a mixture of two components A and B characterized by diffusion coefficients 20 μm^2^s^−1^ and 80 μm^2^s^−1^ and fluorescence lifetimes 4 ns and 2 ns, respectively. (**a**) Influence of the excluded volume; the ideal situation with point-like particles (dotted lines) is compared with the situation in which each particle occupies 4.4% of the detection area (solid lines). (**b**) Influence of electronic dead-time; brightness was 400 kHz per particle, average count rate 394 kHz and electronic dead-time 1 μs. Three cases are compared: correlation functions calculated using all photons (dead-time free, ideal situation, dotted lines), calculated considering the dead-time and using the knowledge which of the simulated molecules emitted each photon (dashed lines) and finally considering dead-time and using FLCS filtering to separate the components (solid lines) (**c**) Influence of detector timing jitter due to intensity-dependent IRF shift on ACFs and CCFs (solid lines). The signal rate dependent jitter was simulated as follows: IRF shift in ps units was 2.4 times the number of photons counts in the last 1 ms. With 41.284 kHz average count rate it means approximately 100 ps average (but fluctuating) shift. Ideal correlation functions calculated using the knowledge which of the simulated molecules emitted each photon instead of FLCS filtering is shown for comparison (dotted lines). Note that the parameters of the shift were chosen arbitrarily and do not simulate any particular type of single photon avalanche diode (SPAD). (**d**) Real experimental data, examples of FLCS CCFs obtained from a mixture of Cy5 and Atto655 [39]; negative CCF amplitude appears even with well matched TCSPC patterns (green solid line) while a deliberate shift of one of the patterns by 60 ps gives rise to a positive cross-correlation (black solid line). FLCS filtered, separated component ACFs of the two compounds are plotted for comparison, in order to demonstrate the relative amplitude of the effects (blue and red lines).The input data were taken from a sample workspace of a commercial software (SymPhoTime, version 5.3.2, PicoQuant, Berlin, Germany, 2010).

First is the trivial effect of excluded volume, which is not specific to FLCS and is inherently present in any form of FCCS experiment. The fraction of effective detection volume occupied by a particle reduces the probability of finding another particle in the detection volume at the same instant. The effect is demonstrated by rather crude simulations shown in Figure 4a. All simulations in Figure 4 were done for a 2-dimensional situation (simulating diffusion in planar structures such as biological membranes). The excluded volume (or rather area in the simulated case) occupied 4.4% of the effective detection area in the simulations; that is comparable to liposomes or polymer beads in the case of 3-dimensional diffusion in a typical confocal detection volume).

Secondly, we have considered the effect of dead time of TCSPC detection. A photon detection event occupies the electronics for some time, during which no other photon can be registered. Simultaneous presence of more molecules within the detection volume increases the fraction of photons reaching the detector “too early upon” a previous photon detection event and therefore not being counted. The fluorescence intensity decay is then distorted by the so-called pile-up effect [37]. Such a decay curve is not well decomposed into decay patterns of individual components, because the pile-up distortion at a given count rate is not uniform: it depends on the shape of the patterns as well as on the fractional intensities emitted with those patterns. Application of the resulting (distorted) FLCS statistical filters then leads to negative CCF amplitudes as shown in Figure 4b. Note that the simulation parameters were selected in order to highlight the photon pile-up; in a real life experiment, better choice of instrumental parameters would considerably decrease the magnitude of the effects.

The third artefact to be considered is the intensity-dependent shift of instrument response function (IRF) typically encountered in SPADs [38]. Because the signal intensity is widely fluctuating during any FCS measurement, the IRF position is dynamically shifting (jittering) and results in smearing of TCSPC histogram, especially visible on its rising edge. That effect naturally influences the statistical filter functions and leads to negative CCFs amplitudes as shown in Figure 4c and also in 4d. Do not confuse the above mentioned dynamic IRF shift during data acquisition with a constant time shift (mismatch) between decay patterns and the total TCSPC histogram. Timing mismatch can have trivial reasons like slow instrumental drifts, or incorrectly matched average photon count rate during pattern acquisition and during the measurement of the mixture. A simple mutual shift of rising edges can be heuristically understood as a time domain analogy to spectral bleed through in dual-colour FCCS that gives rise to false positive cross-correlation (see Figure 4d). Time shift alone is relatively easy to correct by shifting the content of TCSPC histograms channels, nevertheless the effect of dynamic SPAD IRF jitter still remains visible.

The previous paragraphs may result in a false impression that we want to discourage readers from using FLCS for cross-correlation analysis. On the contrary, we think FLCS is a superior method for performing cross-correlation analysis and it overcomes serious problems of dual-colour FCCS connected especially with imperfect overlap of the two excitation and detection volumes. Nevertheless it is good to be aware of the possible artefacts in FLCS CCFs and to reduce them above all by minimizing the pile-up effect (faster detectors and/or electronics, low count rates) and by obtaining as good decay patterns as possible (carefully performed control measurements or elaborate decay model fitting). The third of the sources of anticorrelation in FLCS discussed in the previous paragraph is, in our opinion, the most difficult to avoid and we believe it would deserve a more thorough analysis.

## 5. Alternative Use of FLCS Mathematics

**Figure 5:**
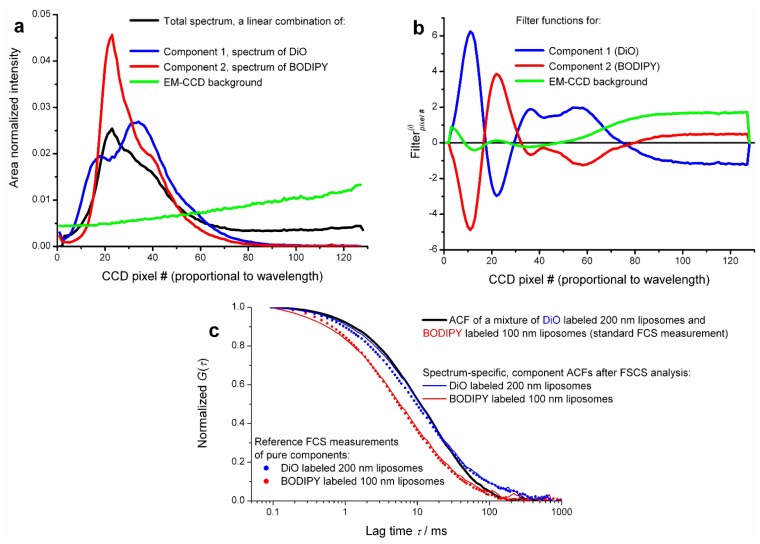
Spectral patterns, associated filter functions and spectrum-specific autocorrelation functions of dye labelled liposomes (unilamellar vesicles). (**a**) Reference spectra (spectral patterns) of 3,3′-dioctadecyloxacarbocyanine perchlorate (DiO) and 4,4-difluoro-1,3,5,7-tetramethyl-4-bora-3a,4a-diaza-s-indacene-8-propionic acid (BODIPY) dyes used for labelling various sized vesicles. Included here is the pattern of the spectral “baseline” caused by fast electron multiplying charge coupled device (EM-CCD) readout as well as the accumulated steady state emission spectrum of the vesicle mixture. Note the complete overlap of the spectra. (**b**) FSCS filter functions calculated for the 3 spectral components. (**c**) Comparison of spectrum-specific ACFs obtained by FSCS of a vesicle mixture and reference ACFs obtained by classical FCS of simple control samples. Although the match (especially for DiO) is not perfect, these results demonstrate a remarkable separation capability of FSCS. Spectral separation of DiO from BODIPY is an extremely difficult case, given the complete overlap of emission spectra.

All the published work so far reviewed in this article employed classical FLCS, where the additional quality enabling separation of ACFs has been obtained from TCSPC, based on short pulsed excitation and measuring the spontaneous fluorescence decay. The mathematics involved in filter calculation is by no means limited to time domain data. For instance the emission spectrum (spectral pattern) identifies a molecule in a same way as does its decay pattern. This idea has been realized by one of us (Aleš Benda) [40] in a FCS format experiment. The method therefore could be called Fluorescence Spectral Correlation Spectroscopy, in short FSCS. Instead of precise timing of single photon detection, the fluctuating signal from a confocal volume is spectrally dispersed by a prism and detected by a single photon sensitive fast electron multiplying charge coupled device (EM-CCD) camera. Both CW and pulsed lasers can be used for excitation. Spectral snapshots are continuously read out using a so called “crop mode” allowing for 62.5 kHz spectral readout. Thus at the end of the measurement there is a long record of spectral snapshots with 16 μs time resolution. This data is post processed so that finally every detected photon is tagged with an arrival time (measured from the start of the experiment) and its spectral detection channel (pixel number). This results in time-tagged spectrally resolved data format, an analogy to time-tagged time resolved data format used in FLCS. By histogramming the spectral channels one obtains the time averaged steady state emission spectrum of the sample. (Again, this is an analogy to the total decay curve in FLCS.) Using reference spectra of the fluorophores as patterns, filter functions are calculated in exactly the same way as in FLCS. Using these filters, spectrum-specific ACFs and CCFs can be calculated. Separation of ACFs as well as CCF analysis of a mixture of two compounds with totally overlapping spectra of different shape has been demonstrated. An example of spectral patterns, filters and spectrum-specific ACFs is shown in Figure 5.

Current limitation of FSCS using EM-CCD detectors is the relatively low time resolution, 16 μs, hindering analysis of fast diffusing species in solution and the very high detector background inevitably stemming from massive detector multiplexing (every pixel acts as a point detector). Fortunately, analysis of both measured and simulated data suggests that for proper data filtering of a two-component mixture as few as 5 to 6 spectral channels may be sufficient. This would allow applying FSCS analysis to data acquired by commercial multichannel spectral single photon counting systems.

A generalization of the FLCS principle was recently proposed by the group of Seidel and denoted by the authors filtered FCS (fFCS) [29,41]. The idea combines the statistical filtering approach developed originally in FLCS with multi-parameter fluorescence detection [42]. The authors have combined FLCS with polarization-resolved detection, thus allowing differences in fluorescence anisotropy to be reflected in the patterns of individual components. However, the idea can be further extended and spectral information included in the patterns as well (for example by splitting the signal spectrally to several TCSPC detection channels). As has been shown by simulations, multi-parameter detection combined with global calculation of statistical filters (using multi-parameter patterns) can lead to good separation of ACFs even in cases when the differences in each of the parameters (such as lifetime, anisotropy, spectrum) between individual components are small and alone give only poor quality of separation of ACFs [29,41].

## 6. Alternative and Related Methods

As has been mentioned already in the seminal FLCS paper [6], fluorescence lifetime information has been used in FCS already in the year 2000 [43]. Time-gated detection of photons (in case of modern instrumentation: time-gate applied to time-tagged photon records) is the simplest approach to resolve signal contributions based on fluorescence lifetime. The simplicity, however, has its price: time-gating means discarding not only the unwanted but also a fraction of the useful signal. For example, discrimination of early photons (simultaneous with the excitation pulse) appears as a viable alternative to get rid of scattered excitation photons. A properly selected time-gate indeed removes all of them, but unfortunately alongside many fluorescence photons, because the discriminated time period corresponds to the highest fluorescence intensity. For instance, let us consider a total IRF duration of 2 ns (typical for a common Si-SPAD detector with IRF FWHM of 400 ps) and a sample with a fluorescence lifetime of 4.1 ns (e.g., ATTO 488 in water). Gating-out the first 2 ns of an experimental decay curve means that only exp (−2 ns/4.1 ns) = 60% of the collected fluorescence photons remain available for correlation. Such a loss can be sometimes compensated by simply longer measurement time and time-gated FCS works reliably when the scattered laser light is the major concern [44]. It cannot remove the effect of detector after pulsing, however, gating is easily combined with a simple after pulsing removal by means of FLCS background subtraction. Unfortunately, gating alone is unable to separate two fluorescence signals excited by the same laser pulse.

In 2010, Ishii and Tahara published a method called Lifetime-Weighted Fluorescence Correlation Spectroscopy, able to resolve lifetime inhomogeneity in an FCS measurement [45]. In this context, inhomogeneity means that distinct molecules in the confocal observation volume feature different or dynamically changing (for example: dependent on conformational changes) fluorescence lifetimes. The idea is to use the measured TCSPC channel number (measured from the onset of laser pulse, interpreted as an instantaneous lifetime sample), as a weighting factor during correlation calculation. Dividing the lifetime weighted correlation curve with conventional (*i.e.*, non-weighted) intensity correlation function reveals the inhomogeneity by deviation from unity. The time dependence of this ratio contains information about the dynamics of lifetime changes, for example about interconversion rates of various molecular conformations.

Two years later the same authors invented another interesting approach [46]. In the context of FLCS it can be described as “FLCS the other way around”. FLCS uses prior information about the decay curves to obtain separate correlation curves for components. The new method of Tahara and Ishii uses the correlation curve as a “map” allowing to pick up photons separated by a selected characteristic lag-time (range), in order to calculate their TCSPC histogram. In other words, the method obtains decay curves of various FCS signal components based on their time-tag (lag time) signatures.

The above two methods are conceptually based on previous work of Yang and Xie [47,48]. Note that the necessary hardware and the primary, recorded data are the same as in the case of FLCS, only the numerical recipes are different. Simple lifetime-weighting [45] is very easy to implement in any FLCS capable software, because only the filter function (*i.e.*, weighting) is affected. However, what appears especially promising is a combined approach when the new method of Tahara and Ishii [46] delivering at least some of the component decay curves is followed by FLCS analysis in order to get their corresponding pure ACFs.

## 7. Conclusions

Over the 11 years of its history, FLCS has become a well established method. It has undergone methodological developments and has found various successful applications in the molecular sciences. The method can be used as an alternative to, as well as improvement of, dual-colour FCCS; it eliminates spectral bleed through and cross-talk. FLCS is a method of choice when the sample is labelled by a single fluorescent dye which exhibits lifetime changes depending either on the local environment or the chemical state of the molecule. The separation principle based on time-domain behaviour overcomes problems with imperfect overlap of excitation and detection volumes hampering any multi-colour confocal experiment. Furthermore, it allows observation of transition kinetics of the fluorophore between its chemical states or molecular microenvironments. FLCS can improve the quality of data in FCS experiments by removal of unwanted effects caused by scattered light and detector artefacts. This is the simplest and most robust feature of this method that can be combined with almost any FCS modality. The idea of statistical filtering can be extended beyond decay patterns as demonstrated here by fluorescence spectral correlation spectroscopy or by the use of global patterns based on multi-parameter fluorescence detection. We hope this review will enlarge the FLCS community and inspire new application ideas the current users may not yet be aware of.
